# Supplementary material for: Cell4D: a general purpose spatial stochastic simulator for cellular pathways
Source: BMC Bioinformatics. 2024 Mar 21;25:121. doi: 10.1186/s12859-024-05739-0 (PMC10956314; doi:10.1186/s12859-024-05739-0)
Supplement: Supplementary file 1 — Additional file 1: Fig. S1. RMSD values of Cell4D particle and bulk diffusion across multiple timescales. [file 12859_2024_5739_MOESM1_ESM.pdf]

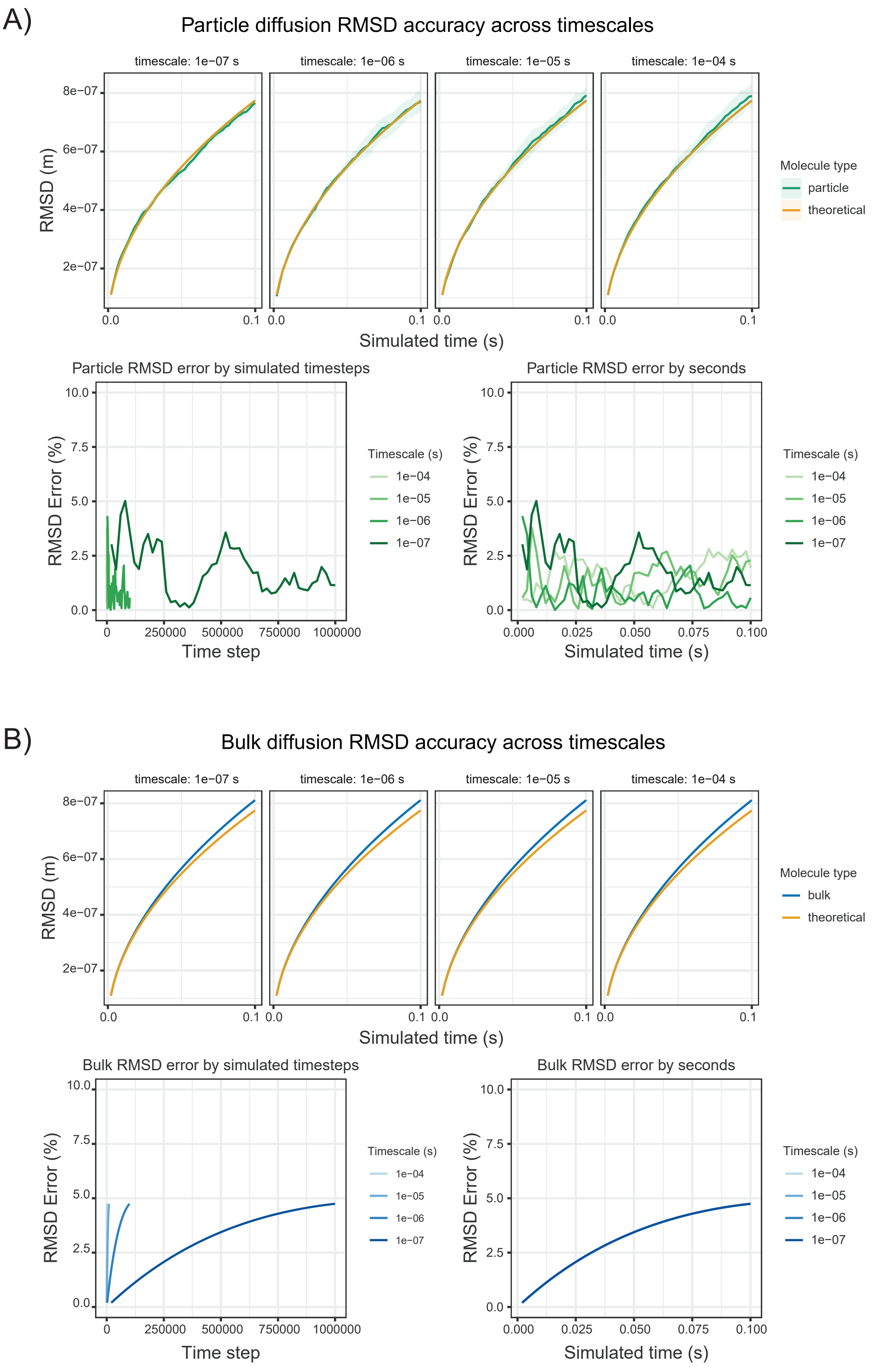

Supplemental Figure 1: RMSD values of Cell4D particle and bulk diffusion across multiple timescales.
A) Upper panel: Comparison of particle RMSD values in green with theoretical Brownian RMSD in orange. Shaded areas indicate standard deviation of particle RMSD across 5 replicates. Lower left panel: The percentage error between simulated and theoretical RMSD at each measured time interval. Lower right panel: Same data as the lower left panel but shown as a function of simulated time length in seconds. B) Upper panel: Comparison of bulk molecule RMSD values in green with theoretical Brownian RMSD in orange, with RMSD remaining the same regardless of the simulation timestep length. Theoretical values were predicted by Fick's laws to examine timestep dependent effects of the bulk diffusion algorithm. Lower left panel: The percentage error between simulated and theoretical RMSD at each measured time interval, shown relative to the number of simulated timesteps. Lower right panel: Same data as the lower left panel but shown as a function of simulated time.
