# Supplementary material for: Cell4D: a general purpose spatial stochastic simulator for cellular pathways
Source: BMC Bioinformatics. 2024 Mar 21;25:121. doi: 10.1186/s12859-024-05739-0 (PMC10956314; doi:10.1186/s12859-024-05739-0)
Supplement: Supplementary file 2 — Additional file 2: Fig. S2. RMSD of Cell4D bulk molecule diffusion and particle diffusion across space scales. [file 12859_2024_5739_MOESM2_ESM.pdf]

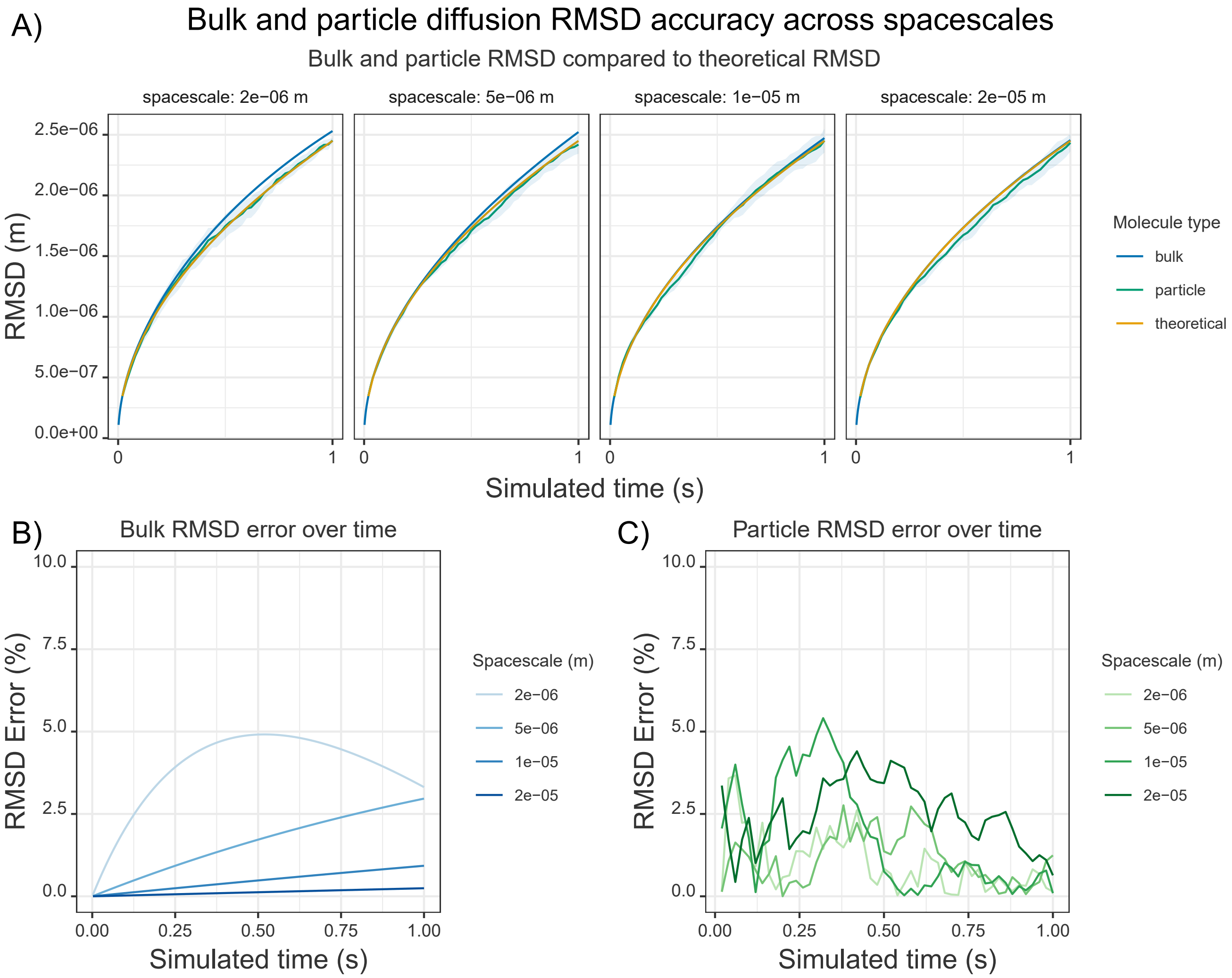

Supplemental Figure 2: RMSD of Cell4D bulk molecule diffusion and particle diffusion across space scales. A) Comparison of bulk molecule RMSD (blue) and particle RMSD (green) across multiple space scales with theoretical Brownian RMSD (orange). B) The RMSD error of bulk molecules as a function of simulation space scale. C) The RMSD error of particle diffusion as a function of simulation space scale. Here, space scale refers to the length represented by each c-voxel. As for the time scale simulations in Supplemental Figure 1, the space scale defined in the simulation has a minimal impact on accuracy of predicted diffusion for both bulk molecules and point particles, with deviations of less than 5% of theoretically determined values.
