# Supplementary figures and images for: Cell4D: a general purpose spatial stochastic simulator for cellular pathways
Source: BMC Bioinformatics. 2024 Mar 21;25:121. doi: 10.1186/s12859-024-05739-0 (PMC10956314; doi:10.1186/s12859-024-05739-0)

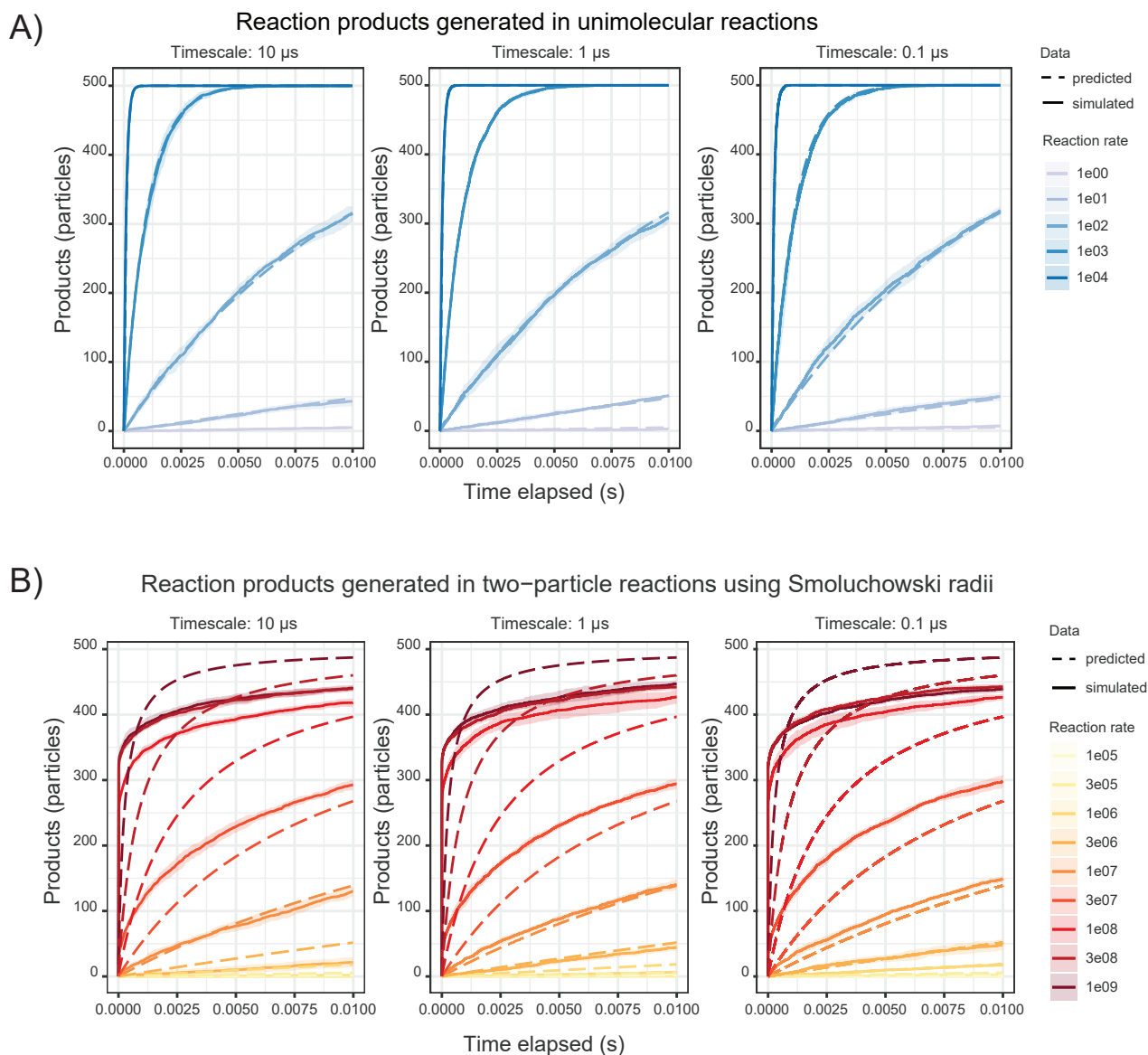

Supplement: Supplementary file 3 — Additional file 3: Fig. S3. Reaction products generated from unimolecular and two-particle bimolecular reactions over time. [file 12859_2024_5739_MOESM3_ESM.pdf]
