# Supplementary material for: Cell4D: a general purpose spatial stochastic simulator for cellular pathways
Source: BMC Bioinformatics. 2024 Mar 21;25:121. doi: 10.1186/s12859-024-05739-0 (PMC10956314; doi:10.1186/s12859-024-05739-0)
Supplement: Supplementary file 4 — Additional file 4: Fig. S4. Summary of bimolecular products over time of bulk-particle reactions using a Smoluchowski-based method to calculate reaction probability. [file 12859_2024_5739_MOESM4_ESM.pdf]

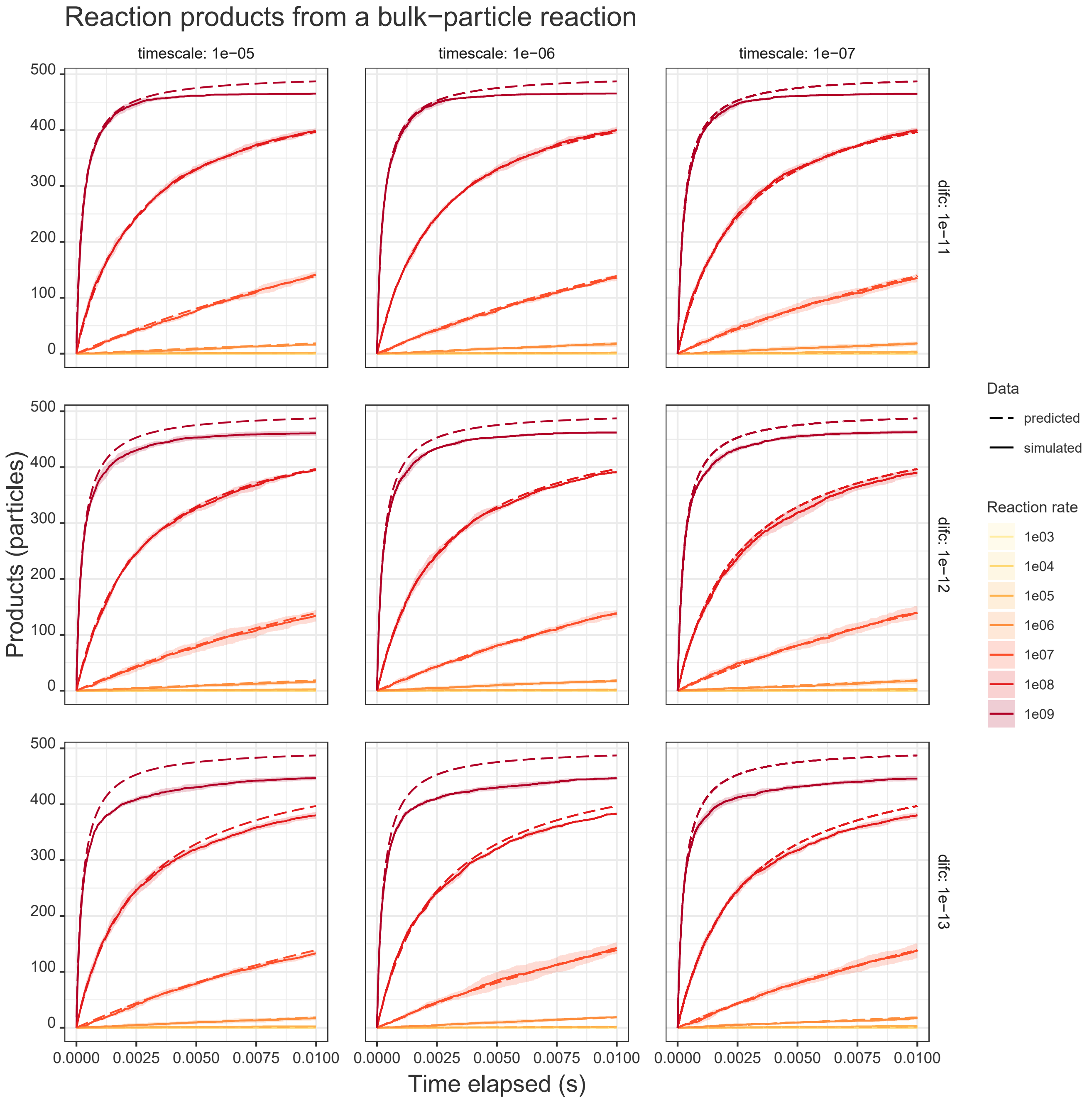


**Supplemental Figure 4: Summary of bimolecular products over time of bulk-particle reactions using a Smoluchowski-based method to calculate reaction probability**.

Dashed lines represent the theoretical yield of products from a bimolecular reaction involving a particle reactant and a bulk molecule reactant over 0.01 seconds. Solid lines represent yield predicted in simulations using Cell4D (mean over 5 replicates). Line colors represent the rate constant of the reaction, with darker colors representing faster rates. Time step lengths of 0.1, 1, and 10 μs were used as simulation parameters, and particle diffusion constants of $1 \times 10^{-11}$ to $1 \times 10^{-13}$ $m^2/s$ were investigated. Little or no product formation is predicted for reaction rates less than $1 \times 10^{6}$ $s^{-1}$. Shaded regions represent the standard deviations.
