# Supplementary material for: Cell4D: a general purpose spatial stochastic simulator for cellular pathways
Source: BMC Bioinformatics. 2024 Mar 21;25:121. doi: 10.1186/s12859-024-05739-0 (PMC10956314; doi:10.1186/s12859-024-05739-0)
Supplement: Supplementary file 5 — Additional file 5: Fig. S5. Summary of two-particle bimolecular products over time using the Andrews-Bray adjusted Smoluchowski method to calculate reaction radii. [file 12859_2024_5739_MOESM5_ESM.pdf]

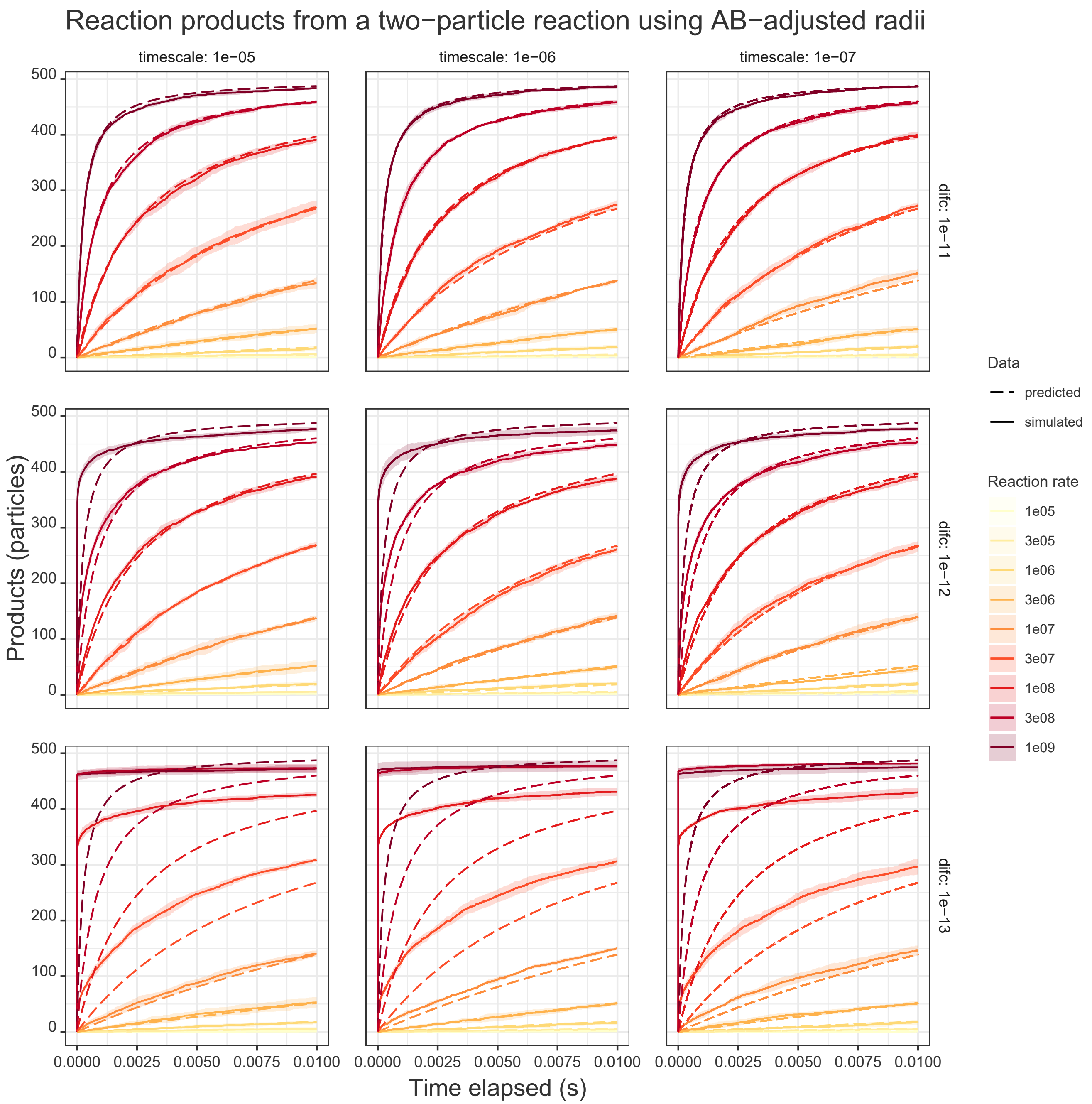

Supplemental Figure 5: Summary of two-particle bimolecular products over time using the Andrews-Bray adjusted Smoluchowski method to calculate reaction radii.
Dashed lines represent the theoretical yield of products from a bimolecular reaction involving two particles over 0.01 seconds. Solid lines represent yield predicted in simulations using Cell4D (mean over 5 replicates). Theoretical yields were obtained from from mass action kinetics using the equation $rate\ -\frac{dA}{dt}\ -k \times A^2$. Simulations used the Andrews-Bray adjusted Smoluchowski method. Line colors represent the rate constant of the reaction, with darker colors representing faster rates. Time step lengths of 0.1, 1, and 10 µs were used as simulation parameters, and particle diffusion constants of 1×10^{-11} to 1×10^{-13} m^2/s were investigated. For reactants with 1×10^{-11} m^2/s diffusion rate, the simulated product curve closely aligns with the theoretical yields at all reaction rates. For diffusion rates of 1×10^{-12} m^2/s, simulations are accurate for reactions up to 1×10^{8} $M^{-1}s^{-1}$. For diffusion rates of 1×10^{-13} m^2/s, simulations perform poorly.
