# Supplementary material for: Cell4D: a general purpose spatial stochastic simulator for cellular pathways
Source: BMC Bioinformatics. 2024 Mar 21;25:121. doi: 10.1186/s12859-024-05739-0 (PMC10956314; doi:10.1186/s12859-024-05739-0)
Supplement: Supplementary file 6 — Additional file 6: Fig. S6. Saturation of calmodulin under varying calcium concentrations in well-mixed or microdomain conditions. [file 12859_2024_5739_MOESM6_ESM.pdf]

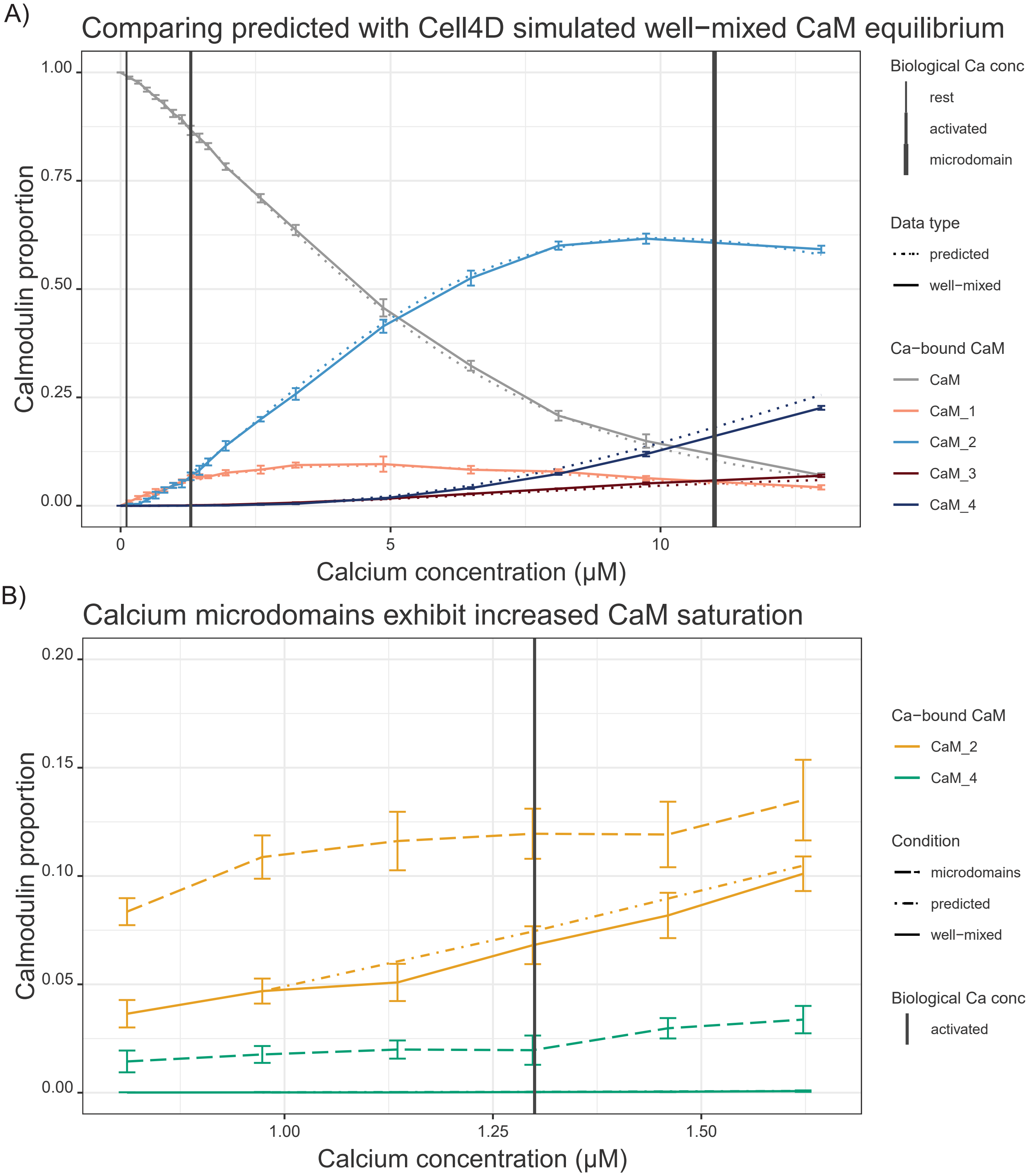

Supplementary Figure 6: Saturation of calmodulin under varying calcium concentrations in well-mixed or microdomain conditions.

A) Proportion of calmodulin saturated with Ca^{2+} ions at equilibrium, with the number suffix denoting the number of Ca^{2+} bound to the calmodulin protein. The dotted and solid lines indicate source of data (Euler-forward deterministic prediction and Cell4D simulations, respectively). Vertical lines mark the average Ca^{2+} concentration within a T cell at rest, activated, and within Ca^{2+} microdomains. Error bars represent standard deviation of Cell4D simulations over 5 replicates. Euler-forward prediction values were calculated at 0.16 μM intervals (50 particles) for a total of 101 data points. B) Comparison of CaM saturation in volumes under well-mixed or microdomain conditions at 0.81, 0.97, 1.14, 1.3, 1.46, and 1.62 μM, with the dotted, dashed, and solid lines representing the predicted well-mixed equilibrium, Cell4D simulated well-mixed results, and Cell4D simulated spatial microdomain conditions. CaM_2 (in yellow) shows the proportion of calmodulin with two bound calcium binding sites, and CaM_4 (green) represents fully saturated calmodulin. Error bars represent standard deviations of Cell4D simulations over 5 replicates.
