# Supplementary material for: Cell4D: a general purpose spatial stochastic simulator for cellular pathways
Source: BMC Bioinformatics. 2024 Mar 21;25:121. doi: 10.1186/s12859-024-05739-0 (PMC10956314; doi:10.1186/s12859-024-05739-0)
Supplement: Supplementary file 7 — Additional file 7: Fig. S7. Comparison of surface CEACAM1 clustering in both Cell4D model variants. [file 12859_2024_5739_MOESM7_ESM.pdf]

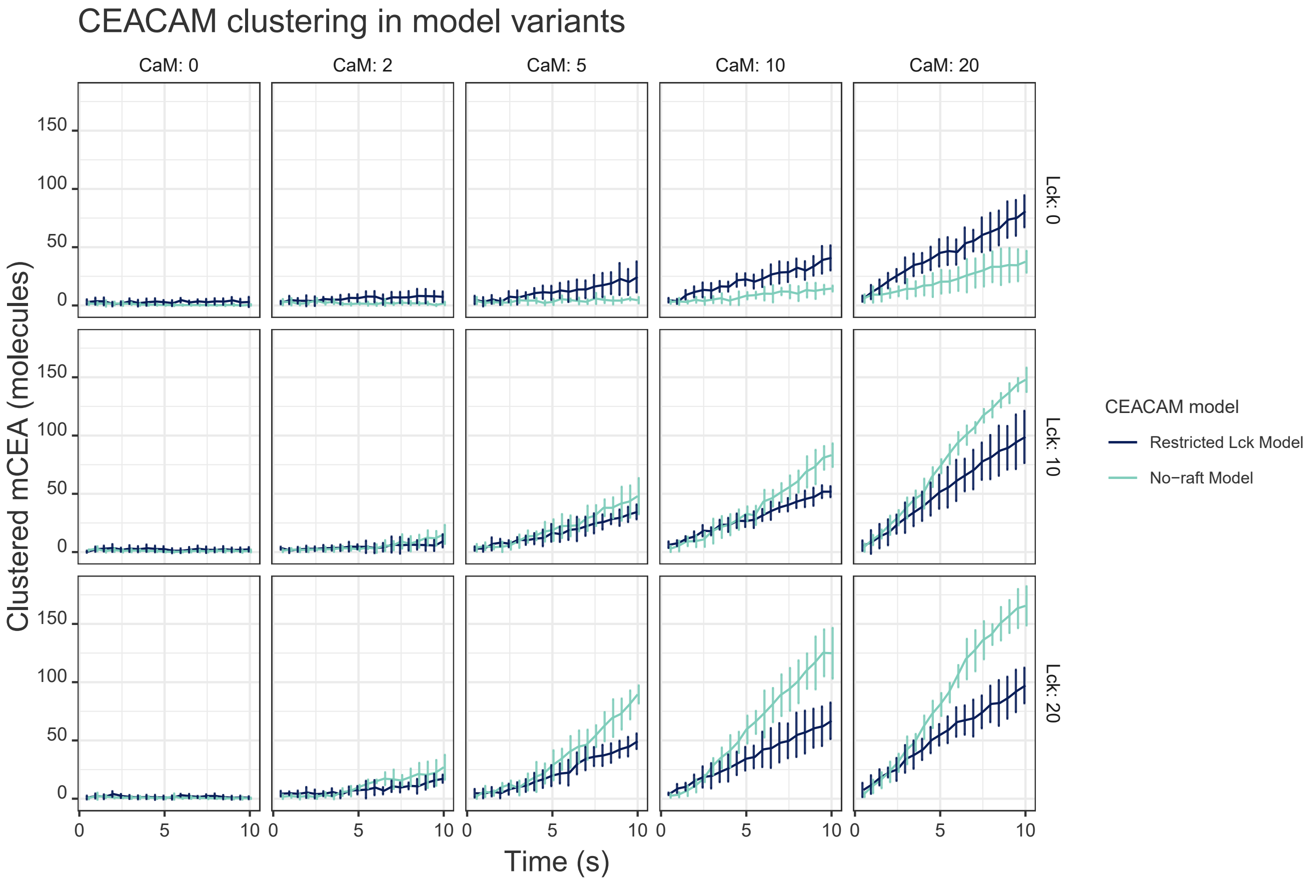

Supplementary Figure 7: Comparison of surface CEACAM1 clustering in both Cell4D model variants.
The amount of clustered membrane monomeric CEACAM1 over 10 simulated seconds is compared between the raft and no-raft models across several calmodulin and Lck concentrations. No clustered CEACAM1 can be found in the calmodulin-absent systems, and the concentration of clustered molecules is positively correlated with calmodulin concentration. In the absence of Lck, the raft model produced more clustered proteins while the opposite is true when Lck is present.
